# Imagination and idealism after the COVID-19 pandemic: the science of healthy ageing

**DOI:** 10.1098/rsos.231102

**Published:** 2024-01-31

**Authors:** Colin Farrelly

**Affiliations:** Political Studies, Queen's University, Kingston, Ontario, Canada K7L 3N6

**Keywords:** ageing, COVID-19, geroscience, idealism and imagination, mRNA vaccines

## Abstract

On 5 May 2023, the World Health Organization declared that COVID-19 no longer constituted a public health emergency of international concern. Medical science must now consider how it ought to recalibrate its imagination and idealism in a post-COVID-19 pandemic world. The fact that *advanced age* was the largest risk factor for COVID-19 mortality and serious illness, as well as for the most prevalent chronic diseases, reveals the urgency and significance of shifting the focus from mitigating each specific pathology risk, one at a time, to targeting biological ageing itself. In his 1910 JAMA Address entitled ‘Imagination and Idealism in the Medical Sciences', Christian Herter made an important distinction between two ways imagination and idealism can be invoked in the medical sciences: (i) humanitarian medicine, which emphasizes the obvious and direct paths of ameliorating human suffering; and (ii) a curiosity-oriented approach which explores pure science and the experimental laboratory. The latter examines the indirect ways of winning, in Herter's words, ‘the citadel’ of health promotion. Herter's reflections on these two contrasting approaches to medicine have significance for both the COVID-19 pandemic and the aspiration to promote the ideal of healthy ageing in the post-COVID-19 pandemic era.

## 1. Introduction

Trust and confidence in the medical sciences is derived, at least in part, from its commitment to the collection and utilization of high-quality empirical evidence [1]. Before any intervention—be it a novel surgical technique, behaviour intervention or medication—makes its way into public health and clinical medicine, empirical evidence concerning its safety and efficacy must be collected and appraised. Thus, a key driver of progress in the medical sciences, which also helps build and sustain public trust and confidence, is the painstaking and tedious task of the systematic collation and synthesis of empirical evidence.

To make substantive progress in the medical sciences, there is also a second important prong, one that involves the exercise of creative and communicative skills quite distinct from those required by the empirical prong charged with the collection and assessment of evidence. This second prong of medical science is responsible for the exercise of *idealism* and *imagination* and provides the general aspirations (e.g. disease control, novel treatment for pathology, etc.) for medical innovation, which thus enable medical researchers to contemplate and pursue novel strategies (e.g. vaccinations, behaviour modification, etc.) to realize those ends and to communicate the significance of medical innovation to policy makers and the general public. The latter is particularly important when it comes to influencing the allocation of public funds for basic scientific research, as well as governmental regulation of biomedical interventions. And, just like the unwavering commitment to empirical evidence, the public's trust and confidence in the medical sciences is also largely contingent upon both the appeal and feasibility of the idealism and imagination it invokes.

Imagination and idealism are the key communicative tools employed in *science advocacy*. From reaping the potential benefits of genome sequencing and editing, to mitigating the harms of climate change, conquering a novel infectious disease and slowing human ageing, public communication about science occurs along a spectrum between science (honesty) and advocacy (effective) [2]. And imagination and idealism provide the cognitive constructs which enable medical researchers to do the research they are passionate about in the first place, and to communicate to policy makers and the general public why this research is imperative.

**Figure 1.**
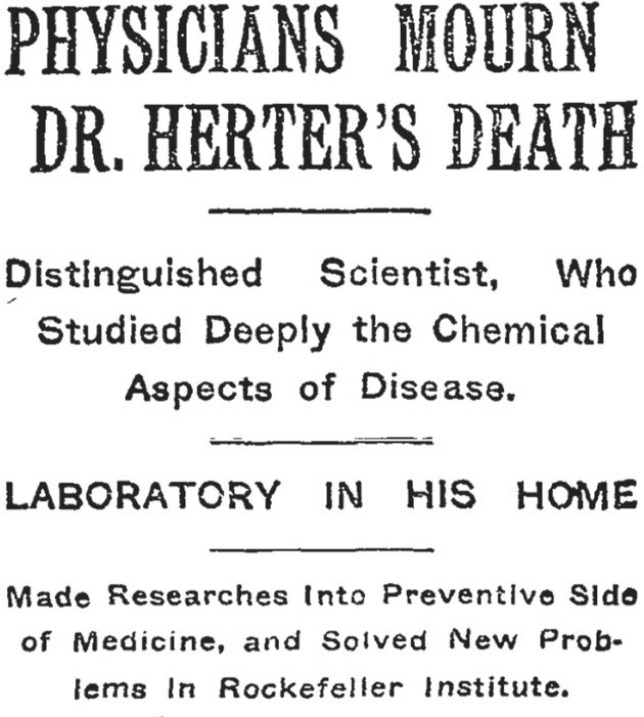
Herter's obituary, *New York Times*, 1910.

In his 1910 JAMA Address entitled ‘Imagination and Idealism in the Medical Sciences', Christian Herter (1865–1910) made an important distinction between two ways idealism and imagination can be invoked in the medical sciences [3]. The first, which he called ‘humanitarian medicine’, focused on the obvious and direct paths of ameliorating human suffering. The second type of medical research was more curiosity-oriented, inspired by the exploration of pure science it emphasized the experimental laboratory as a means to examine the indirect ways of winning, in Herter's words, ‘the citadel’ of health promotion. Herter, who was an active experimentalist, constructed a laboratory in his house [4] and also co-founded the *Journal of Biological Chemistry* in 1905. Tragically he died a few months after the publication of his 1910 JAMA Address (figure 1, obituary headline from *The New York Times* (1910)) [5].

For the past 3+ years, medical researchers, the media, the general public and policy makers have focused their idealism and imagination on managing the COVID-19 pandemic. Herter's JAMA Address has significance for both the COVID-19 pandemic and the aspirations of medical science in the post-COVID-19 pandemic era. The latter ought to prioritize the science of healthy ageing so that older populations can enjoy more years of healthy life (*healthspan*) versus making incremental increases in lifespan by extending the period of time managing multi-morbidity, frailty and disability.

## 2. The COVID-19 pandemic

When the COVID-19 pandemic struck in early 2020 the idealism of humanitarian medicine was primed. SARS-CoV-2 presented novel challenges for both the evidence-based prong of medical science, as well as the exercise of idealism and imagination. There were many unknowns, such as the lethality of the virus and how it was transmitted. Researchers sought to quickly fill this lacuna of knowledge with the rapid publication of an unprecedented number of scientific studies about the virus, its various mutations, the impact of different proposed mitigation strategies (e.g. face mask mandates, school closures, etc.) and the short- and long-term health impacts of infection. The COVID-19 pandemic was the first global pandemic that the scientific publishing industry faced and an estimated 1.5 million articles were published in just 2020 [6].

Humanitarian medicine during the first year of the pandemic focused on the obvious strategy of *behaviour modification* to try to create safer environments which would either slow, or perhaps (it was initially hoped) even eliminate, the spread of SARS-CoV-2. A diverse range of ideals and justifications for the pandemic restrictions were put forth by public health officials and experts, ranging from the aspiration to eliminate the virus (zero-COVID) [7], to avoiding overwhelming healthcare systems (flattening the curve) [8], to those who advocated for a return to somewhat normal life (for at least the majority of the population) with a strategy of ‘focused protection’ for the more vulnerable [9].

Herter's emphasis on the importance of curiosity-driven medicine, which advances more slowing via the experimental method and its exploration of pure science, proved to be a key turning point in the efforts to mitigate the public health threat from SARS-CoV-2. The initial research that made mRNA vaccines for COVID-19 possible was undertaken by hundreds of scientists, dating back to the 1960s [10], long before SARS-CoV-2 even existed, let alone was a global health threat. For decades, the basic scientific research on mRNA advanced without any clear or obvious medical application.

## 3. The idealism and imagination behind the mRNA vaccines

In May 2020, US President Donald J. Trump launched ‘Operation Warp Speed’ [11], priming the idealism that safe and effective vaccines would be developed within months, making the stringent mitigation measures of social isolation obsolete. And by December 2020 *The New England Journal of Medicine* published the advanced access results of both mRNA vaccines for SARS-CoV-2 [12,13]. And while mutations of the virus reduced the effectiveness of the vaccines to stop transmission, COVID-19 vaccines reduced the risks of serious illness and death and are estimated to have saved 14.4 million lives between 8 December 2020 and 8 December 2021 [14]. And the estimate of deaths averted per vaccine administered was notably higher in high-income and upper-middle-income countries, in part because of greater access to more efficacious mRNA vaccines.

Over half a century of experimental science, going back to experiments in the 1960s with liposomes and mRNA laid the initial foundations for the medical research needed to develop mRNA vaccines in the year 2020. And for decades, this research did not have any clear medical application. And yet the research continued and, eventually, found its way to a significant public health intervention.

The COVID-19 pandemic has had a significant impact on the intellectual culture and aspirations of medical science. On 5 May 2023, the World Health Organization declared that COVID-19 no longer constituted a public health emergency of international concern.^1^ And thus it is an important time for medical science to consider how it ought to recalibrate its imagination and idealism in a post-COVID-19 pandemic world. Perhaps the obvious way forward, advocated strongly by infectious disease experts and the World Health Organization [15], is to prioritize the idealism of preparing for the next infectious disease outbreak, priming the imagination necessary to conceive of novel ways to prevent, and respond more effectively to, infectious diseases.

But the fact that *advanced age* (age greater than 60 years) was the largest risk factor for COVID-19 mortality [16] and serious illness, as well as for the most prevalent chronic diseases (like heart disease and cancer) reveals the urgency and significance of shifting the focus from mitigating each specific pathology risk, one at a time, to targeting biological ageing itself. The idealism of healthy ageing is arguably the most important ideal for medical science today given the fact that unprecedented numbers of humans are expected to survive into late life this century, reaching an estimated 3.1 billion people age 60 or older by the year 2100 [17].

## 4. The ideal(s) of healthy ageing

**Figure 2.**
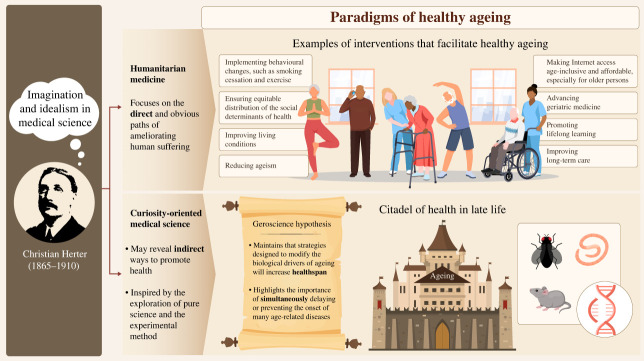
Imagination and idealism in medical science.

The United Nations and World Health Organization have designated 2021–2030 as the ‘decade of healthy ageing’ [18,19]. However, it is useful to distinguish between two different ways idealism and imagination are invoked with respect to discussions of healthy ageing (figure 2). And these two ways cohere with the two categories of medicine Herter identified in 1910, with respect to humanitarian medicine and curiosity-oriented/experimental science.

Humanitarian medicine's aspirations focus on the obvious and direct ways of ameliorating the suffering and vulnerabilities of late life. One obvious example of humanitarian medicine to emphasize in the post-pandemic era is the importance of interventions to promote social connectedness among older persons. Older persons living in long-term care and assisted living facilities were already, pre-pandemic, vulnerable to the harms of isolation and the prolonged social restrictions of the pandemic exacerbated this problem [20–23]. The WHO's goals for the decade of healthy ageing include other, similar, aspirations of humanitarian medicine- age-friendly environments, combating ageism and eliminating food insecurity, promoting lifelong learning and age-inclusive and affordable access to the Internet for older persons.

**Figure 3.**
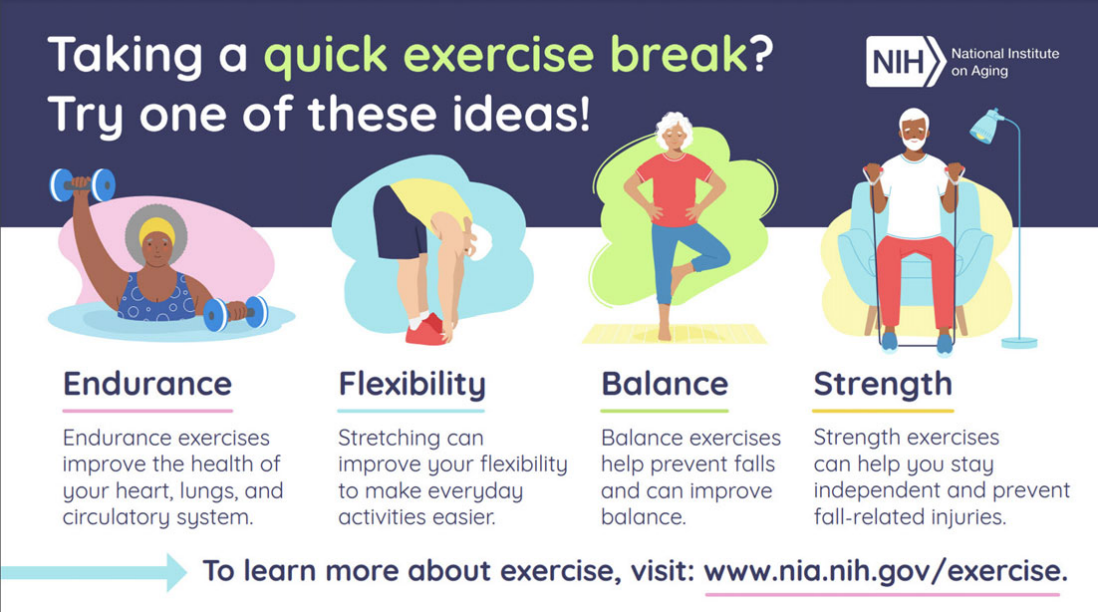
National Institutes of Health (NIH) exercise infographic.

Other obvious aspects of humanitarian medicine's prescriptions for healthy ageing involve lifestyle changes (e.g. physical exercise) which can increase healthspan. The National Institute on Aging (NIA), for example, share an infographic on ‘Four Types of Exercise and Physical Activity’ (figure 3, credit National Institute on Aging) to help promote health in late life.^2^ These include endurance, flexibility, balance and strength. The US Department of Health and Human Services' Guidelines on physical activity also prescribes regular aerobic activity and muscle-strengthening activities [24]. However, in 2020 only one in four adults aged 18 and over met these exercise guidelines [25].

While there is a general consensus on the issue that exercise can increase healthspan, the issue of the impact exercise has on the mechanisms of ageing is one that divides many scientists that study the biology of ageing. Some contend that exercise is a potent anti-ageing and anti-chronic disease medicine, and should be examined further as a potential senolytic medicine (which targets senescent cells) for ageing and various diseases [26]. Austad contends that, while exercise has been shown to increase mean longevity in both rats and people, it is not generally considered an intervention that slows ageing because it does not increase maximal survival (though he notes many in the field are beginning to rethink this) [27]. After decades of public health disseminating the knowledge about the health benefits of exercise it is clear that poor compliance (especially in the elderly population) makes this challenging to apply [28]. Like trying to combat the risks from infectious diseases by relying solely on the behavioural interventions of hand washing, wearing face masks and isolating the infected (versus having the benefits of vaccines), to substantively improve the quality of life of older persons medical innovation that develops pharmacological interventions to slow ageing will be necessary, in addition to encouraging more exercise across the lifespan.

As Herter noted in 1910, the limitation of humanitarian medicine is that it does not entice the idealism or imagination needed to search for the indirect ways of promoting health. In fact, it often opposes such creative thinking, taking it to represent a violation of, or potential threat to, the priority of humanitarian medicine. Humanitarian medicine would not, for example, propose targeting the rate of biological ageing itself as that is not (as of yet, at least) a direct and obvious route to health promotion. Thus one ideal of healthy ageing, when construed through the lens of humanitarian medicine, engages the imagination of improved living conditions for older persons and behaviour modifications (e.g. exercise, healthy diet, smoking cessation, etc.) than can increase healthspan.

The more bold aspiration of rate (of ageing) control [29] comes from experimental science rather than humanitarian medicine. The driving force behind research into modifying the mechanisms of ageing was research on caloric restriction (CR) and longevity in a variety of laboratory animals. Unlike physical exercise, CR is able to delay ageing processes that increase both mean and maximum lifespan versus primarily increasing healthspan [30]. This scientific research, detailed in the following section, aroused the idealism and imagination encapsulated in what the field of geroscience now refers to as the *Geroscience Hypothesis*, a hypothesis that builds upon nearly a century of research on the biology of ageing and experimentation in altering the lifespan of a variety of laboratory organisms. The Geroscience Hypothesis maintains that strategies designed to modify the biological drivers of ageing will not only slow the progression of biological ageing, but will also prevent or delay the onset of multiple chronic diseases [27].

## 5. The Geroscience Hypothesis: a century in the making

Experimental research in the early twentieth century began examining the impact of dietary restriction on growth and longevity in laboratory rats [31–33]. By 1935, the empirical evidence established that CR in rats increased lifespan [34]. CR is defined as a decrease of 30% to 60% *ad libitum* feeding without malnutrition [30]. And research on CR in mice, conducted during the 1970s and 1980s, demonstrated conclusively that CR had a major impact on ageing by preventing/delaying the incidence of most age-related diseases and pathologies in rodents [35]. Short-lived species like rodents, worms and fruit flies were the ideal species to examine for laboratory experiments on the biology of ageing because of their short lifespan and the low cost of such experiments.

Research on the impact of CR on ageing and longevity was not ‘pathology’ research. There was no obvious, clinically relevant application driving a century of experimental research on ageing in rodents and invertebrates. Geroscience is perhaps the exemplar example of the curiosity-oriented type of research Herter championed in 1910, guided by an interest in pure science and slowly, but surely, edging its way closer to ‘the stream of medical utility’ once the ability to genetically manipulate laboratory organisms accelerated knowledge about the mechanisms of ageing. And this slow and steady progress has faced its sceptics and setbacks. When describing the progress of experimental science Herter makes an analogy with an ‘ever-broadening and deepening river’, that faces currents which are ‘expressive of certain doubts and errors that fringe all progress' ‘into the somewhat sluggish stream of medical utility’. As Austad notes [18], one major setback for geroscience was a 1999 study [36] that reported that a targeted mouse mutation increased lifespan by 30%, but the finding was never independently validated until 15 years later and could not be replicated [37].

One potential, though likely unfeasible, intervention for altering the rate of ageing in humans concerns dietary restriction. This includes CR but also some type of (intermittent and periodic) [38] fasting. The empirical evidence on both the potential feasibility for such interventions to alter biological ageing in humans, and to do so safely, is something researchers have only recently begun to explore and is still ongoing. Long-term CR is too burdensome (as well as potentially dangerous) of an intervention to expect people to comply with, and there are no studies examining the long-term impact of CR on human healthspan or lifespan. Intermittent fasting also faces practical challenges, such as the abundance of food and marketing of food in developed nations, as well as people experiencing hunger, irritability and a reduced ability to concentrate during periods of food restriction (at least for the first month) [39].

CALERIE^3^ (Comprehensive Assessment of Long-term Effects of Reducing Intake of Energy) is an NIA-supported clinical trial and the first study to focus specifically on the effects of sustained CR in humans. CALERIE 2 tested effects of a 2-year randomized clinical trial in which 220 non-obese adults were compared after being separated into one of two groups—those on a 25% CR diet (compared with their pre-test daily caloric intake) and those that continued to consume their regular amount of calories. However, the average CR actually achieved in the treatment group was roughly half the prescribed dose of 25%, and this was over a relatively short (from the adult lifespan perspective) period of time (2 years). The scientific value of studies on CR and fasting in humans lays not with the potential to prescribe such burdensome interventions as feasible, long-term, population-wide preventative medicine strategies (though some researchers do advocate some form of dietary restriction as a feasible ageing intervention), but rather to help shed light on how CR in humans can impact healthspan and longevity so that drugs that mimic the effects of dietary restriction to delay the onset of disease, frailty and disability in late life can be safely developed.

## 6. ‘Anti-ageing’ drugs

Like trying to combat the risks of infectious diseases with only the (time-limited) behaviour modifications of regular hand washing, wearing face masks and isolation of the infected, to prescribe only behaviour interventions (e.g. smoking cessation, exercise and diet) to combat the risks of chronic disease, frailty and disability in late life reflects the constrained aspirational vision of humanitarian medicine. It was not until into the twenty-first century that translating the discoveries made by genetically manipulating ageing in invertebrates and mice was translated into potential pharmacological/pharmaceutical interventions that could be tested in humans [27]. A number of compounds have now been tested in mice for their positive effects on lifespan and ageing. These include metformin [40] and rapamycin [41]. The former has been safely used in humans for decades as a pharmacological intervention to help control type 2 diabetes. And rapamycin is a compound that has also been used as a drug for humans to help prevent the rejection of transplanted organs for patients undergoing organ transplant. One of the most effective strategies for developing an applied gerontological intervention is the repurposing of existing drugs that have been approved by the FDA for human therapy, as such drugs are likely to have low risks, so that small benefits from retarding ageing might provide an acceptable risk/benefit ratio [42].

One major challenge the field of geroscience faced was determining how to assess a compound's impact on the mechanisms of ageing given that senescence is a complex mixture of processes (versus a monolithic process) and senescence and disease have overlapping biological consequences [43]. The field has united around the identification of the so-called ‘hallmarks of ageing’ which attempt to identify and categorize the cellular and molecular hallmarks of ageing [44]. The initial hallmarks of ageing included genomic instability, telomere attrition, epigenetic alterations, loss of proteostasis, deregulated nutrient sensing, mitochondrial dysfunction, cellular senescence, stem cell exhaustion and altered intercellular communication. Additional hallmarks of ageing continue to be identified [45]. By identifying specific biomarkers for the mechanisms of ageing, *gerotherapeuthics*—drugs that target pathways involved in ageing with the aim of reducing the burden of ageing-related diseases and increasing lifespan and healthspan [46] may be successfully developed. Drug development (e.g. the design of clinical trials) was originally designed for targeting specific diseases versus ageing itself. To accommodate the idealism of healthy ageing inspired by experimental medical research, regulatory organizations must establish ageing as a target and outcome as this would open up the ageing research field to the pharmaceutical industry for investment, expansion and clinical application [47].

## 7. Conclusion

Medical science progresses when both the empirical prong of evidence-based medicine and the idealism and imagination of innovative thinking and feasible aspirations work *in tandem* to reveal new ways to prevent and target disease and/or promote health. As the intense public attention to COVID-19 begins to wane, medical science ought to focus on the significant, and feasible, aspiration of healthy ageing. The humanitarian medicine articulation of this ideal will champion the obvious and direct ways to ameliorate some of the health vulnerabilities of late life, such as a more equitable distribution of the social determinants of health which influence rate of ageing [48], physical exercise, a healthy diet and geriatric medicine.

But insights from experimental gerontology also reveal novel ways healthy ageing may be realized, pointing to imaginative strategies that transcend the traditional focus on improving living conditions and behaviour change. One important lesson to be learned from the COVID-19 pandemic is that relying only on the latter is of limited value and faces significant feasibility constraints. Social isolation and wearing face masks are, at best, temporary versus viable long-term preventative measures for mitigating the health risks of SARS-CoV-2. The same may be true for the ideal lifestyle prescriptions of humanitarian medicine and chronic disease, given the reality of multi-morbidity in advanced age and the low compliance rates with ideal lifestyle prescriptions.

Furthermore, if innovation in the medical sciences only pursues the strategy of trying to tackle each chronic disease, one at a time, through developing novel treatments for each disease (all types of cancer, heart disease, Alzheimer's, etc.), it is a costly and Herculean task yielding diminishing health dividends for older populations. Such a strategy may delay the age of death from specific diseases, but that should not be equated with improving the *quality of life* of older persons.

It is worth recalling that, more than half a century ago, US President Richard Nixon invoked the idealism of a ‘cancer-free’ future when he announced a ‘war on cancer’ in 1971. And while improvements have been made in delaying the average age of death from cancer, unfortunately there has not been a single cure for any of the 200+ types of cancer that cause mortality in humans. Cancer remains, as it was when a war against cancer was first declared, the second leading cause of death in the USA [49,50].

As Herter noted over a century ago, to ‘win the citadel’ of health promotion attention must also be given to the slow, and gradual advances made in experimental science. The idealism of drug development that targets the mechanisms of ageing, thereby increasing the healthspan, is very distinct from the current disease-specific approach of the medical sciences. The longest lived humans, such as centenarians and supercentenarians who achieve their longevity without enduring onerous physical exercise or dietary regimes, provide a feasible ideal of what might constitute healthy longevity. Such an ideal transcends what healthy lifestyle alone could potentially offer most of the population. The development of drugs that slow the ageing process—thereby extending the healthspan and compressing disease, frailty and disability at the end of life—would arguably constitute the most significant advance in public health this century, given the global prevalence of age-related diseases.

## Data Availability

This article has no additional data.

## Footnotes

1 https://www.who.int/news/item/05-05-2023-statement-on-the-fifteenth-meeting-of-the-international-health-regulations-(2005)-emergency-committee-regarding-the-coronavirus-disease-(covid-19)-pandemic.

2 https://www.nia.nih.gov/health/four-types-exercise-can-improve-your-health-and-physical-ability.

3 https://calerie.duke.edu/.
